# No-patch 23-gauge vitrectomy under topical anesthesia: A pilot study

**DOI:** 10.4103/0301-4738.77038

**Published:** 2011

**Authors:** Satyen Deka, Harsha Bhattacharjee, M J Barman, Kruto Kalita, Sunil Kumar Singh

**Affiliations:** Department of Vitreoretina Services, Sri Sankaradeva Nethralaya, Beltola, Guwahati-781 028, India

**Keywords:** Topical anesthesia, transconjunctival, 23-gauge vitrectomy

## Abstract

A pilot study was designed to evaluate the safety and efficacy of 23-gauge vitrectomy under topical anesthesia. Five eyes of five patients underwent 23-gauge sutureless vitrectomy under topical anesthesia with a pledget soaked in 0.5% proparacaine hydrochloride anesthetic, for vitreous hemorrhage (four eyes), epiretinal membrane (one eye). Subjective pain and discomfort were graded using a visual analogue chart from 0 (no pain or discomfort) to 4 (severe pain and discomfort). At the end of surgery no patch was applied and patients were given dark glasses. Patients underwent an immediate postoperative assessment, followed by next day and one week postoperative evaluation. Four patients had Grade 0 pain during the surgery. One patient had Grade 1 pain during the placement and withdrawal of the micro cannulas. The surgical outcomes were favorable. 23-gauge vitrectomy under topical anesthesia is safe and effective in selected cases. Further study is recommended to validate the outcome of this study.

Intraocular surgeries have been performed under retrobulbar, peribulbar and topical anesthesia.[1,2] Traditionally, vitreoretinal surgeries are performed under general anesthesia or peribulbar anesthesia. Recently, Eckardt improved the transconjunctival sutureless technique introducing the 23-guage Tranasconjunctival Sutureless Vitrectomy (TSV) system.[3] There are reports of conventional 20-gauge vitrectomy, 23-gauge and 25-gauge sutureless vitrectomy surgeries being done under topical anesthesia.[4,5] This brief report describes the results of 23-gauge vitreoretinal surgery, performed under topical anesthesia without sedation.

## Materials and Methods

Five eyes of five consecutive patients underwent 23-gauge vitrectomy under topical anesthesia. Surgical indications were epiretinal membrane (ERM) causing metamorphopsia in one case and non-resolving vitreous hemorrhage in four cases. Out of four vitreous hemorrhage cases, two cases were of Eales’ disease and two of branch retinal vein occlusion (BRVO) origin. Cases were selected based on:

- Clinical judgment and surgeon’s (SD) experience that expected surgical time should be minimal in all those cases,
- Patients behavior during the examination procedure. Out of five patients two were hypertensive.

**Figure 1:**
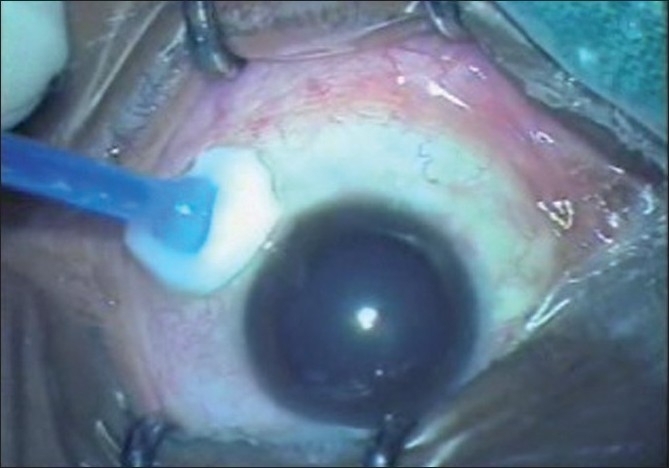
Application of pledget soaked in 0.5% proparacaine hydrochloride at the proposed sclerotomy site

**Figure 2:**
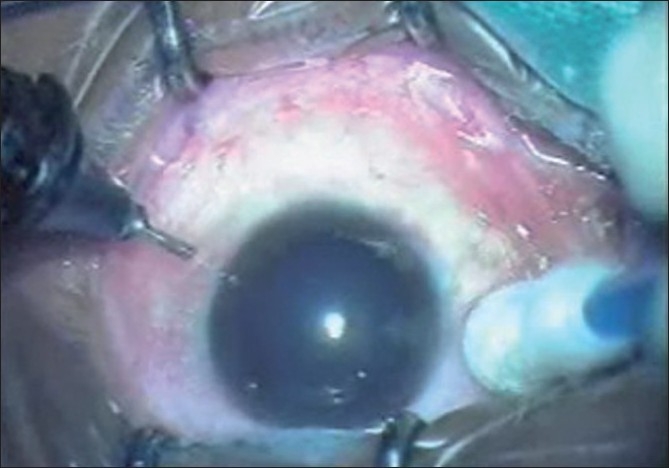
Insertion of 23-gauge trocar and cannula

**Figure 3:**
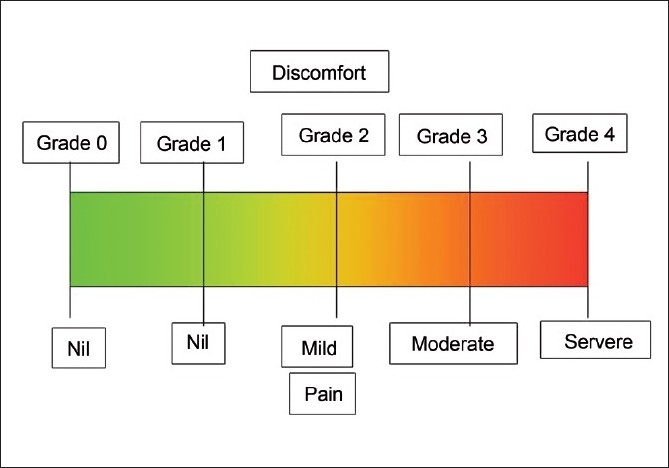
Visual analogue scale

The three other patients did not have any appreciable systemic disease. Both the hypertensive patients were on amlodepine 5 mg daily dose and were well controlled at the time of surgery. After achieving maximum pupillary dilatation with 1% tropicamide and 5% phenylephrine, the conjunctival cul-de-sac was first anesthetized with a drop of 0.5% proparacaine hydrochloride (Paracain 0.5% eye drops). The eye and the surrounding area were then cleaned and painted with povidone iodine 5%. After draping, the eye was kept open using the universal speculum. Pledget soaked in 0.5% proparacaine hydrochloride anesthetic drop was placed at the three proposed sclerotomy sites for one minute just before entry [Fig. 1]. Using the 23-gauge trocar and cannula system (Alcon Labs, USA), three transconjunctival sclerotomy entries were made, 3.5 mm posterior to the limbus at the inferotemporal, superonasal and superotemporal locations, taking care to stretch the overlying conjunctiva with a sterile cotton bud. The trocar and cannula were pushed through the sclera parallel to the limbus initially at an oblique angle and then perpendicularly into the vitreous cavity, and the trocar was removed [Fig. 2]. The infusion was maintained through the inferotemporal sclerotomy. The core vitrectomy was performed without base excision using the dual dynamic drive technology on the accurus machine (Alcon Labs, USA). After completion of vitrectomy, endolaser photocoagulation was performed in four cases using 532 nm frequency doubled Nd:YAG laser through a 23-gauge laser endoprobe (Iridex Corporation, USA) as they were secondary to proliferative retinal vasculopathies. A 23-gauge forceps (Alcon Labs, USA) was used for membrane- peeling in one case. Fluid/air exchange and SF6 injection was done in the ERM case where persistent retinal wrinkling was present even after the ERM was removed. In all cases routine scleral indentation was performed to check the sclerotomies before removing the cannulas. At the end of the procedure, the superotemporal and superonasal cannulas were removed first, followed by the removal of the inferotemporal cannula with the infusion line, after closing the infusion. A drop of gatifloxacin eye drops and cyclopentolate hydrochloride 1% was instilled into the cul-de-sac and the eye was left open without any patch. The patients were given protective spectacles to wear. Immediately after the surgery each patient was shown a five-point subjective pain and discomfort visual analogue scale: 0 = no pain or discomfort, 1 = no pain but mild discomfort, 2 = mild pain and discomfort, 3 = moderate pain and discomfort and 4 = severe pain and discomfort [Fig. 3] and then asked to grade the level of pain and discomfort during the surgical procedure. All patients were started on prednisolone acetate eye drops every 2 h and tapered over three weeks, gatifloxacin eye drops every 2 h for one week. The patients were evaluated next day and at one week. None of the patients required any sedation intraoperatively or in the postoperative period.

## Results

**Figure 4:**
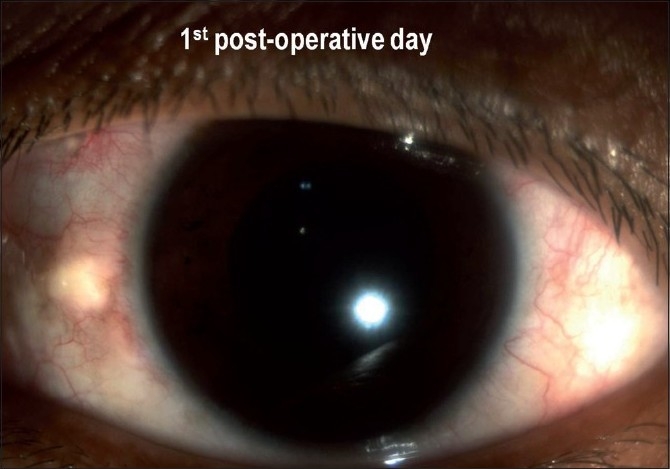
Postoperative first day slit-lamp photograph showing minimal inflammation

**Figure 5:**
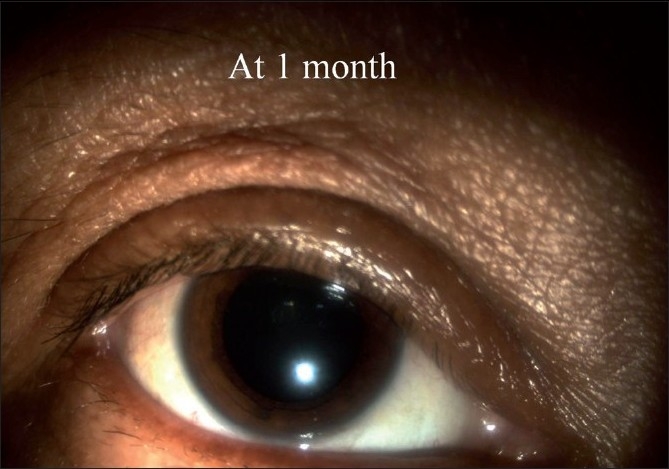
Postoperative one month slit-lamp photograph showing quiet eye

The mean age of the patients was 48 years (28 to 62 years). Out of five eyes three were phakic eyes. All patients had visual improvement at one week and one month postoperative follow-up [Table 1]. Minimal subconjunctival escape of gas was noted in one case; that resolved spontaneously and there was no hypotony. There was no incidence of trauma to the crystalline lens in the phakic eyes. Four patients had Grade 0 pain throughout the procedure. One patient reported Grade 1 pain during insertion and withdrawal of microcannula. None of the patients required oral analgesics in the postoperative period. Intraocular pressures recorded (all cases) at Day one, at Week one, and at Month one follow-up were normal (mean 18 + 2.4 mm Hg, range14 to 20 mm Hg). There was minimal intraocular inflammation in all cases [Fig. 4]. The operated eye was quiet at one month follow-up [Fig. 5].

**Table 1:** Visual outcome

Pre-op BCVA	BCVA at 1 week	BCVA at 1 month	Complications
20/60	20/40	20/20	Nil
FCCF	20/40	20/30	Nil
20/400	20/80	20/20	Nil
FCCF	20/30	20/25	Nil
20/400	20/200	20/60	Subconjunctival gas

BCVA: Best corrected visual acuity, FCCF: Finger counting close to face

## Discussion

Conventional 20-gauge vitrectomies have been successfully performed under topical anesthesia with sedation.[4] Most of these reports have recorded Grade 2 level of pain and discomfort during cauterization of scleral bed, during incision of sclerotomy, suturing of sclerotomy and conjunctiva. 25-gauge vitrectomies have been successfully done under topical anesthesia without sedation using anesthetic-soaked pledget at the site of sclerotomies.[5] The pledget delivery of anesthetic has the added advantage of prolonged delivery of the anesthetic to the areas where the sclerotomies are planned thereby contributing to reduced pain and discomfort during the procedure.[6]

Theocharis *et al*., indicate that topical anesthesia could be considered an alternative to other anesthetic procedures in 25-g and 23-g vitrectomies.[7] The 23-gauge vitrectomy system relies on the trocar and cannula system for the sclerotomies. Conjunctival peritomy is not required and there is no contact of instruments with sclera or pars plana. 23-gauge vitrectomy is ideal for topical vitreoretinal surgeries in selected cases. The added advantage of topical anesthesia was that patients could be instructed to move the eye in the required direction intraoperatively whenever necessary as there was no akinesia. All complications related to local anesthesia can be averted.[8]

All patients had favorable visual and surgical outcome. Postoperative ocular inflammation was minimal and none of the patients complained of pain in the immediate postoperative period. 23-gauge sutureless vitrectomy under topical anesthesia is safe and effective in selected cases only. Further study is recommended to validate the outcome of this study.
